# A Story of PA/BSA and Biomarkers to Diagnose Pulmonary Hypertension in Patients with Severe Aortic Valve Stenosis—The Rise of IGF-BP2 and GDF-15

**DOI:** 10.3390/jcdd10010022

**Published:** 2023-01-05

**Authors:** Joseph Kletzer, Stefan Hecht, Susanne Ramsauer, Bernhard Scharinger, Reinhard Kaufmann, Jürgen Kammler, Jörg Kellermair, Kaveh Akbari, Hermann Blessberger, Clemens Steinwender, Klaus Hergan, Uta C. Hoppe, Michael Lichtenauer, Elke Boxhammer

**Affiliations:** 1 Department of Internal Medicine II, Division of Cardiology, Paracelsus Medical University of Salzburg, 5020 Salzburg, Austria; 2 Department of Radiology, Paracelsus Medical University of Salzburg, 5020 Salzburg, Austria; 3 Department of Cardiology, Kepler University Hospital, Medical Faculty of the Johannes Kepler University Linz, 4020 Linz, Austria; 4 Department of Radiology, Johannes Kepler University Hospital Linz, 4020 Linz, Austria

**Keywords:** aortic valve stenosis, biomarker, computed tomography, pulmonary hypertension, PA/BSA, TAVR

## Abstract

(1) Background: Currently, echocardiography is the primary non-invasive diagnostic method used to screen patients with severe aortic valve stenosis (AS) for pulmonary hypertension (PH) by estimating systolic pulmonary artery pressure (sPAP). Other radiological methods have been a focus of research in the past couple of years, as it was shown that by determining the pulmonary artery (PA) diameter, prognostic statements concerning overall mortality could be made in these patients. This study compared established and novel cardiovascular biomarkers with the PA/BSA value to detect PH in patients with severe AS. (2) Methods: The study cohort comprised 188 patients with severe AS undergoing transcatheter aortic valve replacement (TAVR), who were then divided into two groups based on PA/BSA values obtained through CT-angiography. The presence of PH was defined as a PA/BSA ≥ 16.6 mm/m^2^ (n = 81), and absence as a PA/BSA < 16.6 mm/m^2^ (n = 107). Blood samples were taken before TAVR to assess cardiovascular biomarkers used in this study, namely brain natriuretic peptide (BNP), cardiac troponin I (cTnI), high-sensitive troponin (hsTN), soluble suppression of tumorigenesis-2 (sST2), growth/differentiation factor 15 (GDF-15), heart-type fatty acid-binding protein (H-FABP), insulin-like growth factor binding protein 2 (IGF-BP2), and soluble urokinase-type plasminogen activator receptor (suPAR). (3) Results: Patients with a PA/BSA ≥ 16.6 mm/m^2^ showed significantly higher levels of BNP (*p* = <0.001), GDF-15 (*p* = 0.040), and H-FABP (*p* = 0.007). The other investigated cardiovascular biomarkers did not significantly differ between the two groups. To predict a PA/BSA ≥ 16.6 mm/m^2^, cut-off values for the biomarkers were calculated. Here, GDF-15 (*p* = 0.029; cut-off 1172.0 pg/mL) and BNP (*p* < 0.001; cut-off 2194.0 pg/mL) showed significant results. Consequently, analyses of combined biomarkers were performed, which yielded IGF-BP2 + BNP (AUC = 0.721; 95%CI = 0.585–0.857; *p* = 0.004) as the best result of the two-way analyses and GDF-15 + IGF-BP2 + BNP (AUC = 0.727; 95%CI = 0.590–0.864; *p* = 0.004) as the best result of the three-way analyses. No significant difference regarding the 1-year survival between patients with PA/BSA < 16.6 mm/m^2^ and patients with PA/BSA ≥ 16.6 mm/m^2^ was found (log-rank test: *p* = 0.452). (4) Conclusions: Although PA/BSA aims to reduce the bias of the PA value caused by different body compositions and sizes, it is still a controversial parameter for diagnosing PH. Combining the parameter with different cardiovascular biomarkers did not lead to a significant increase in the diagnostic precision for detecting PH in patients with severe AS.

## 1. Introduction

Patients suffering from severe aortic stenosis (AS) regularly have concomitant pulmonary hypertension (PH), leading to increased all-cause mortality in surgical and interventional AS repair [1,2]. Invasive right heart catheterization has been used in pre-operative/pre-interventional diagnostics to assess the additional risk posed by PH. This practice has been largely abandoned in recent years due to new findings regarding the evaluation of PH through echocardiographic measurements such as systolic pulmonary artery pressure (sPAP) [3,4]. Studies have shown that sPAP can not only be used to accurately diagnose PH, but also as an independent risk factor for early and long-term mortality, as well as length of hospital stay [5,6]. However, there is no definitive consensus in the literature concerning the cut-off value of sPAP defining the presence of PH. Current guidelines and studies give sPAP values ranging from ≥40 mmHg to ≥50 mmHg, leading to a lack of standardization [3,4,5,6,7,8,9].

Additionally, CT angiography is utilized to gain information about the great arteries and aortic valve annulus of every patient undergoing TAVR. As per current European Society of Cardiology (ESC) guidelines [10], CT imaging may be used to assess PH in a non-invasive manner [11,12]. Pulmonary artery diameter/body surface area (PA/BSA), with a cut-off value of 16.6 mm/m^2^, was used in this study to possibly diagnose PH.

In addition to imaging, several biomarkers have been found to correlate with important cardiovascular diseases. New biomarkers such as soluble suppression of tumorigenesis-2 (sST2), growth/differentiation factor 15 (GDF-15), heart-type fatty acid-binding protein (H-FABP), insulin-like growth factor binding protein 2 (IGF-BP2), and soluble urokinase-type plasminogen activator receptor (suPAR) are starting to be mentioned together with classical biomarkers such as brain natriuretic peptide (BNP) and cardiac troponin I (cTnI) because of their potential for an adequate diagnosis of cardiovascular diseases, including myocardial infarction [13,14] or heart failure [15,16].

As the literature investigating the predictive value of biomarkers regarding the concurrence of severe AS and PH is rare, this study aims to compare PA/BSA obtained through CT angiographic imaging with biomarkers, to assess the presence of PH in patients with severe AS before TAVR in a non-invasive manner.

## 2. Materials and Methods

### 2.1. Study Population

Data of 188 patients with severe, primary degenerative AS who were supposed to undergo the TAVR procedure were analyzed at Paracelsus Medical University Hospital Salzburg and Kepler University Hospital Linz between 2016 and 2018. Local ethics committees at Paracelsus Medical University Salzburg (415-E/1969/5-2016) and Johannes Kepler University Linz (E-41-16) reviewed and approved the study protocol. All study participants consented in writing to the parameters of this study.

### 2.2. Transthoracic Echocardiography

Guidelines of the European Society for Cardiology were used to classify severe AS, defined as AV Vmax (maximal velocity over aortic valve) of 4.0 m/s, AV dpmean (mean pressure gradient over aortic valve) ≥ 40 mmHg, and an aortic valve area ≤ 1.0 cm^2^. Simpson’s method was used to calculate left ventricular ejection fraction (LVEF). Valve regurgitations of mitral, aortic, and tricuspid valves were classified as minimal, mild (I), moderate (II), and severe (III) using spectral and color Doppler images. Continuous wave Doppler was used over the tricuspid valve to obtain maximum tricuspid regurgitant jet velocity (TRV). Using the formula 4 × TRV^2^ + estimated right atrial pressure (RAP), the pulmonary artery pressure (PAP) was calculated. Estimated RAP corresponds to the central venous pressure, which is determined by the inferior vena cava (IVC) diameter (Table 1):

Finally, sPAP was calculated using the simplified Bernoulli equation (4 × TRV^2^) + RAP, which was then used to determine PH in accordance with the current literature [3,4,5,7,8,9]. To perform transthoracic echocardiography, common devices (iE33 and Epiq 5; Philips Healthcare) were used.

### 2.3. CTA Protocol and Measurement of PA Diameter for PH Assessment

**Figure 1:**
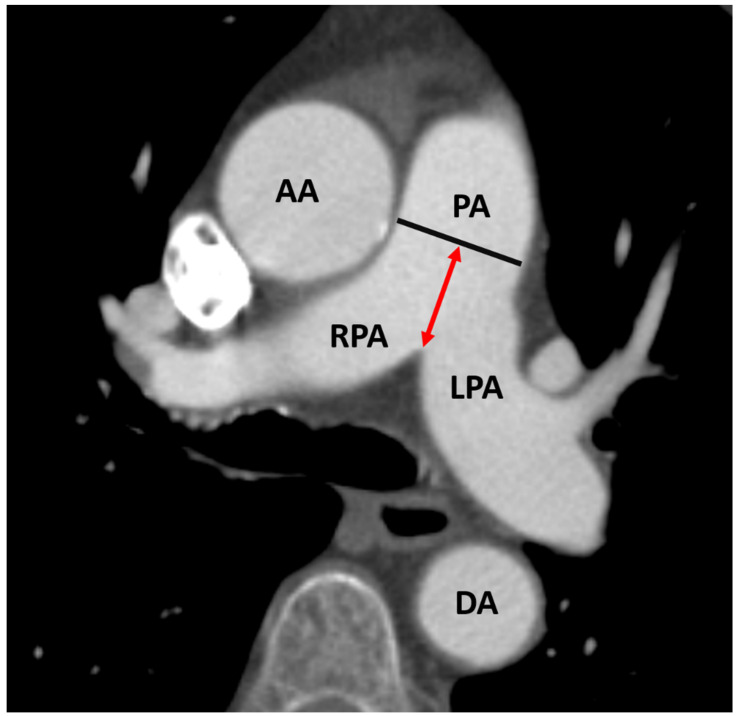
Measurement of pulmonary artery diameter (black line) on axial CT. PA: pulmonary artery; red double-headed arrow: distance between bifurcation of the pulmonary artery and level of measurement within pulmonary artery; RPA: right pulmonary artery; LPA: left pulmonary artery; DA: descending aorta; AA: ascending aorta.

Before TAVR, aortic annulus size, aortic anatomy, and vascular access were evaluated using ECG triggered CT-angiography (second-generation, multidetector 128-slice dual source CT; Somatom Definition AS+, Siemens Healthcare, Erlangen, Germany) of the aorta and femoral arteries. A 100 mL bolus of non-ionic iodinated contrast media followed by 70 mL saline solution injected at 3.5–5 mL/s were used to visualize the vasculature. Two radiological investigators who had no prior knowledge of the patients’ clinical information analyzed the images on a stationary workstation (Impax, Agfa-Gevaert, Mortsel, Belgium). The diameter of the pulmonary artery (PA) was measured within 3 cm of the bifurcation, as can be seen in Figure 1. The quotient of PA/BSA was generated using the DuBois formula (BSA = 0.007184 × Height^0.725^ × Weight^0.425^).

### 2.4. Biomarker Analysis

Biomarker analysis was performed using samples obtained one day before TAVR, as well as 3 and 12 months after the procedure in an outpatient setting using a vacuum-containing system. The collection tubes were centrifuged to separate plasma from blood components and then frozen at −80 °C. The samples of all 188 patients were measured at similar time points under the same conditions. To avoid bias in the biomarker analyses, patients with end-stage renal disease or requiring dialysis and patients with previous myocardial infarction or coronary intervention with drug-eluting stent implantation <1 month before TAVR were excluded in advance.

Enzyme-linked immunosorbent assay (ELISA) kits (sST2: DY523, GDF-15: DY957, H-FABP: DY1678, IGF-BP2: DY674, suPAR: DY807, R&D Systems, Minneapolis, MN, USA) were used to quantify plasma levels of GDF-15, H-FABP, IGF-BP2, and suPAR. Serum samples and standard protein were put onto the ELISA plate wells (Nunc MaxiSorp flat-bottom 96 well plates, VWR International GmbH, Vienna, Austria) and incubated for two hours, as per the manufacturer’s instructions. After they were treated with Tween 20/PBS solution (Sigma Aldrich, USA), a biotin-labeled antibody was added and incubated for a further 2 h. They were then washed, and streptavidin horseradish peroxidase solution was added. After adding tetramethylbenzidine (TMB; Sigma Aldrich, St. Louis, MO, USA), the resulting color reaction could be measured at an optical density of 450 nm using an ELISA plate-reader (iMark Microplate Absorbance Reader, Bio-Rad Laboratories, Vienna, Austria).

The selection of biomarkers was based on extensive literature research as well as preliminary work of our own research group: sST2 has already been described as a potentially valuable biomarker for risk stratification of PH in TAVR patients [17]. IGF-BP2 is considered a general risk predictor in patients with severe AS and TAVR [18]. High plasma levels of suPAR have been associated with increased postoperative mortality and complications after surgical aortic valve replacement [19]; increased mortality after TAVR was found in patients with elevated GDF-15 levels [20]. Finally, H-FABP as a relevant biomarker for acute myocardial injury was shown to be decreased in the course after TAVR, so the hypothesis was raised that falling H-FABP levels after TAVR are associated with improvement of the hemodynamic situation [21].

### 2.5. Statistical Analysis

Statistical analysis was performed using SPSS (Version 25.0, SPSSS Inc., Armonk, NY, USA). GraphPad Prism (Version 8.0.0, GraphPad Software, San Diego, CA, USA) as well as SPSS were used to create figures.

The estimated sample size was calculated using the following formula, where *z* stands for confidence interval, *p* for the sample proportion, and *ε* for the margin of error:

(1) $$\frac{z^{2}*p\left(1-p\right)}{\varepsilon^{2}}$$

This was repeated using different confidence levels and margin errors (Figure S1).

To test variables for normal distribution, the Kolmogorov–Smirnov test was used. If metric data were normally distributed, they were expressed as mean ± standard deviation (SD) and analyzed using the unpaired Student’s *t*-test. For non-normally distributed metric data, the Mann–Whitney U-test was used; median and interquartile range (IQR) were used to convey the data. Categorical data were compared using the chi-square test.

To determine a cut-off value for PA/BSA, area under the receiver operating characteristics (AUROC) curves with area under the curve (AUC) and subsequent Youden Index (YI) analyses were performed at different sPAP values (sPAP 40–45–50 mmHg). Biomarker plasma levels were then statistically compared based on two groups (“No PH”: PA/BSA < cut-off and “PH”: PA/BSA ≥ cut-off; cut-off = 16.6 mm/m^2^) using AUROC analyses and YI analyses for each examined biomarker.

Twofold and threefold biomarker combinations by first completing a binary logistic regression for each combination and then submitting the obtained values to an AUROC analysis. All possible combinations of the named biomarkers (sST2, GDF-15, H-FABP, IGF-BP2, suPAR, BNP, hsTN, cTnI) were considered in this study.

A Kaplan–Meier curve with a log-rank test was generated to compare the 1-year survival between patients with a PA/BSA above and below 16.6 mm/m^2^.

Statistical significance was set to a *p*-value ≤ 0.050.

## 3. Results

### 3.1. Sample Size Calculation

Using the aforementioned formula to calculate, it was assessed that in this study, for a 95% confidence interval/5% margin error, a sample size of 385 subjects would have been needed. For a 90% confidence interval/10% margin error, the sample size necessary was calculated to be 69 patients. Averaging the four calculated versions of the estimated sample size in this study equals 206 patients, which is in an acceptable range to our cohort of 188 patients. Supplementary Figure S1 provides further detail and summarizes the sample size calculation.

### 3.2. Study Cohort

Of the 188 patients included in this study, 107 patients (56.9%) had a PA/BSA ratio measured through CT angiography below 16.6 mm/m^2^, which we defined as “No PH”. The remaining 81 patients (43.1%) showed a PA/BSA ratio higher or equal to 16.6 mm/m^2^, which was equated to them having PH.

### 3.3. Baseline Characteristics

In Table 2, the baseline characteristics of the overall cohort, as well as the characteristics of the two groups defined as PA/BSA < 16.6 mm/m^2^ and PA/BSA ≥ 16.6 mm/m^2^ are listed and compared using the statistical methods laid out above.

Most of the study population was male (52.70%), and the percentages for the PA/BSA < 16.6 mm/m^2^ and the PA/BSA ≥ 16.6 mm/m^2^ groups differed significantly (60.7% vs. 42.0%; *p* = 0.011). The average age of the cohort was calculated to be 82.8 years ± 4.9 years. The two groups also differed in weight (76.0 kg ± 15.5 kg vs. 71.0 kg ± 12.8 kg; *p* = 0.021), height (168.6 cm ± 8.7 cm vs. 164.7 cm ± 9.0 cm; *p* = 0.003), and STSScore mean (2.4 ± 1.0 vs. 3.5 ± 1.8; *p* = 0.005). Concerning clinical characteristics, a difference in mean LVEF (57.5% ± 9.4% vs. 53.4% ± 12.4%; *p* = 0.015) and sPAP (38.5 mmHg ± 14.3 mmHg vs. 49.0 mmHg ± 14.8 mmHg; *p* = <0.001) between the two groups was seen. Other basic characteristics did not show any significant difference between the two groups investigated.

### 3.4. AUROC Results—sPAP vs. PA/BSA Ratio

**Figure 2:**
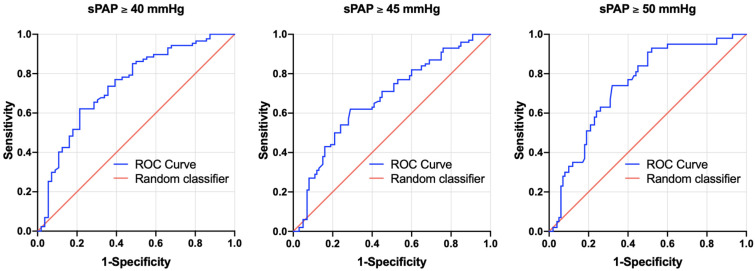
AUROC curves of PA/BSA values for the prediction of sPAP ≥ 40, 45, and 50 mmHg with concerning cut-off values, Youden Indexes, sensitivity, and specificity. sPAP: systolic pulmonary artery pressure.

With the goal of calculating a cut-off value for the PA/BSA ratio to accurately diagnose PH, an AUROC analysis was performed, which is shown in Figure 2 and Table 3.

For sPAP ≥ 40 mmHg, a PA/BSA cut-off of 16.53 mm/m^2^ was calculated (AUC = 0.741; 95% CI = 0.656–0.826; *p* = <0.001; sensitivity = 0.62; specificity = 0.79; YI = 0.41). Moreover, 16.65 mm/m^2^ was determined to be the cut-off for sPAP ≥ 45 mmHg (AUC = 0.672; 95% CI = 0.584–0.761; *p* = <0.001; sensitivity = 0.62; specificity = 0.71; YI = 0.33). Finally, sPAP ≥ 50 mmHg led to a PA/BSA cut-off of 16.65 mm/m^2^ (AUROC = 0.742; 95% CI = 0.658–0.826; *p* = <0.001; sensitivity = 0.74; specificity = 0.68; YI = 0.42).

A rounded PA/BSA of 16.6 mm/m^2^ was used as a cut-off value in this study based on these calculations.

### 3.5. Biomarker Concentrations in the Study Cohort

**Figure 3:**
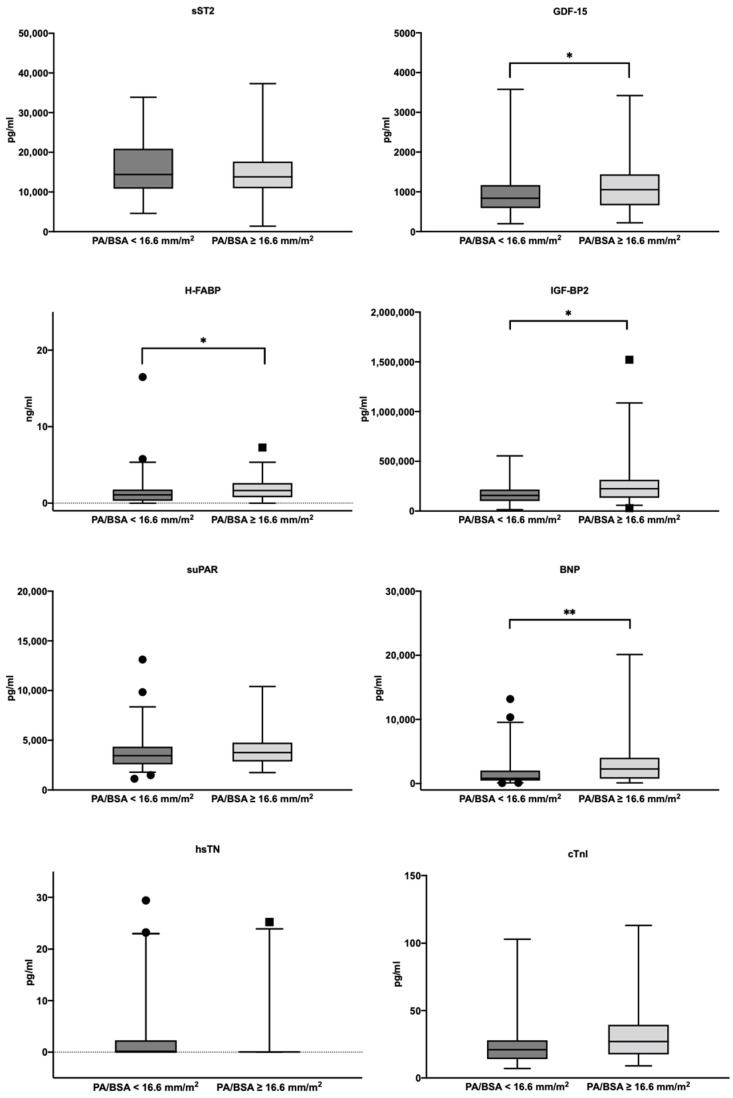
Serum concentrations of sST2, GDF-15, H-FABP, IGF-BP2, suPAR, BNP, hsTN, and cTnI in patients with a PA/BSA value ≥ 16.6 mm/m^2^ and with a PA/BSA value < 16.6 mm/m^2^; ** *p* ≤ 0.010; * *p* ≤ 0.050. PA: pulmonary artery; BSA: body surface area; BNP: brain natriuretic peptide; cTnI: cardiac troponin I; hsTN: high-sensitive troponin; sST2: soluble suppression of tumorigenesis-2; GDF-15: growth/differentiation factor-15; H-FABP: heart-type fatty acid-binding protein; IGF-BP2: insulin-like growth factor binding protein 2; suPAR: soluble urokinase-type plasminogen activator receptor.

A comparison of the cardiovascular biomarker concentrations regarding the PA/BSA cut-off value determined can be found in Figure 3.

A significant difference in the median biomarker concentrations between patients with a PA/BSA < 16.6 mm/m^2^ and patients with a PA/BSA ≥ 16.6 mm/m^2^ was seen in BNP (823.1 pg/mL with IQR 1557.3 pg/mL vs. 2270.5 pg/mL with IQR 3263.7 pg/mL; *p* = <0.001), hematocrit (39.1% with IQR 5.3% vs. 37.4% with IQR 7.7%; *p* = 0.013), hemoglobin (13.1 g/dL with IQR 2.4 g/dL vs. 12.6 g/dL with IQR 2.9; *p* = 0.011), GDF-15 (840.1 pg/mL with IQR 577.7 pg/mL vs. 1055.2 pg/mL with IQR 778.9 pg/mL; *p* = 0.040), H-FABP (1.1 ng/mL with IQR 1.5 ng/mL vs. 1.6 ng/mL with IQR 1.9 ng/mL; *p* = 0.007), and IGF-BP2 (156,947.1 pg/mL with IQR 115,637.5 pg/mL vs. 225,232.1 pg/mL with IQR 180,916.1 pg/mL; *p* = 0.005). The remaining cardiovascular biomarkers did not show any significant difference between the two PA/BSA groups.

### 3.6. AUROC Results—PA/BSA vs. Singular Biomarkers

**Figure 4:**
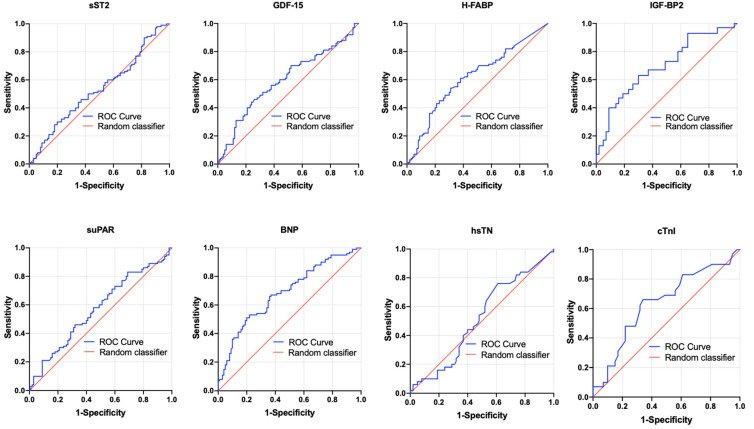
AUROC curves of sST2, GDF-15, H-FABP, IGF-BP2, suPAR, BNP, hsTN, and cTnI for prediction of a PA/BSA value ≥ 16.6 mm/m^2^ with concerning cut-off values, Youden Indexes, sensitivity and specificity. BNP: brain natriuretic peptide; cTnI: cardiac troponin I; hsTN: high-sensitive troponin; sST2: soluble suppression of tumorigenesis-2; GDF-15: growth/differentiation factor-15; H-FABP: heart-type fatty acid-binding protein; IGF-BP2: insulin-like growth factor binding protein 2; suPAR: soluble urokinase-type plasminogen activator receptor.

To evaluate the potential of biomarkers to predict PA/BSA ≥ 16.6 mm/m^2^ in patients with severe AS before undergoing TAVR, AUROC and YI analyses were again performed on each of the biomarkers (sST2, GDF-15, H-FABP, IGF-BP2, suPAR, BNP, hsTN, cTnI). Figure 4 and Table 4 depict the results of these analyses.

With *p*-values ≤ 0.05, significant results were found for GDF-15 (AUROC = 0.595, 95%CI = 0.509–0.680, *p* = 0.029, sensitivity = 0.44, specificity = 0.76, YI = 0.20) with a cut-off value of 1172.0 pg/mL and for BNP (AUROC = 0.684, 95%CI = 0.604–0.764, *p* = <0.001, sensitivity = 0.51, specificity = 0.80, YI = 0.32) with a cut-off value of 2194.0 pg/mL. IGF-BP2 (AUROC = 0.694, 95%CI = 0.570–0.818, *p* = 0.05, sensitivity = 0.63, specificity = 0.70, YI = 0.33) with a cut-off value of 198,440.4 pg/mL also showed promising results with statistical significance just barely present (*p* = 0.050).

sST2, H-FABP, suPAR, hsTN, and cTnI did not show any significant results in their AUROC analyses.

### 3.7. AUROC Results—PA/BSA vs. Dual-Biomarker Combinations

**Figure 5:**
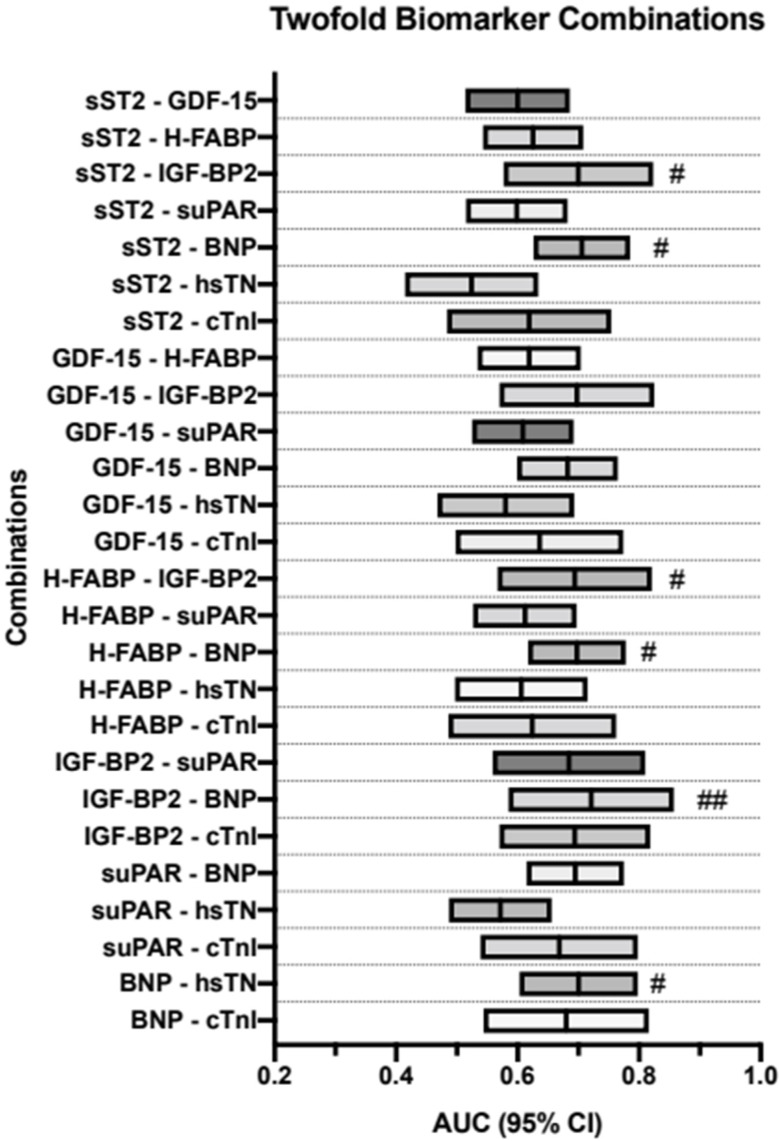
Two-way biomarker combinations with corresponding AUROC analyses and diagnostic strength; # YI ≥ 35; ## YI ≥ 40. BNP: brain natriuretic peptide; cTnI: cardiac troponin I; hsTN: high-sensitive troponin; sST2: soluble suppression of tumorigenesis-2; GDF-15: growth/differentiation factor-15; H-FABP: heart-type fatty acid-binding protein; IGF-BP2: insulin-like growth factor binding protein 2; suPAR: soluble urokinase-type plasminogen activator receptor; YI: Youden Index.

As stated previously, the biomarkers were then combined using a binary logistical regression and AUROC and YI analyses were performed on the resulting data. Firstly, they were performed on all possible dual-biomarker combinations of the biomarkers used in this study up to this point. Results can be seen in Table 5 and are visualized in Figure 5.

Most of the combinations showed significant *p*-values. The most promising combinations in these two-way analyses were sST2 + BNP (AUROC = 0.706, 95%CI = 0.626–0.785, *p* = <0.001, sensitivity = 0.71, specificity = 0.67, YI = 0.38), H-FABP + BNP (AUROC = 0.698, 95%CI = 0.617–0.778, *p* = <0.001, sensitivity = 0.78, specificity = 0.62, YI = 0.39) and IGF-BP2 + BNP (AUROC = 0.721, 95%CI = 0.484–0.857, *p* = 0.004, sensitivity = 0.68, specificity = 0.73, YI = 0.41).

### 3.8. AUROC Results—PA/BSA vs. Triple-Biomarker Combinations

**Figure 6:**
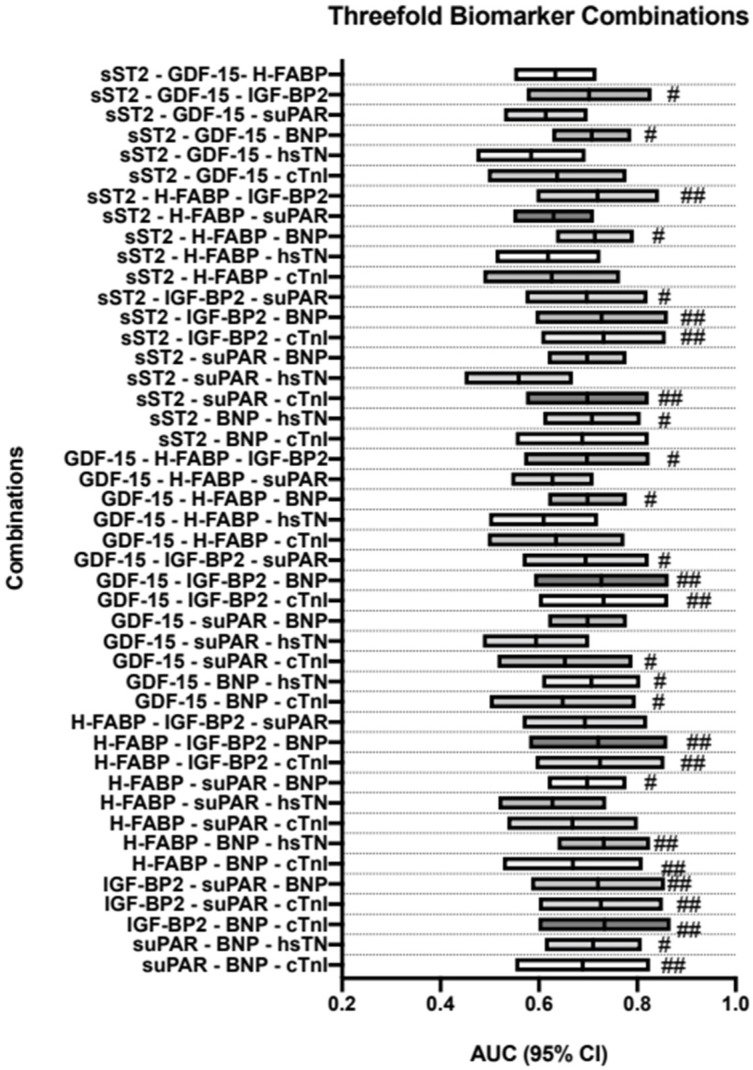
Three-way biomarker combinations with corresponding AUROC analyses and diagnostic strength; # YI ≥ 35; ## YI ≥ 40. BNP: brain natriuretic peptide; cTnI: cardiac troponin I; hsTN: high-sensitive troponin; sST2: soluble suppression of tumorigenesis-2; GDF-15: growth/differentiation factor-15; H-FABP: heart-type fatty acid-binding protein; IGF-BP2: insulin-like growth factor binding protein 2; suPAR: soluble urokinase-type plasminogen activator receptor; YI: Youden Index.

All possible three-way combinations of these biomarkers were then analyzed in the same manner as the dual combinations. Findings are depicted in Table 6 and are visualized in Figure 6.

Combinations of particular note were GDF15 + IGF-BP2 + cTnI (AUROC = 0.732, 95%CI = 0.600–0.863, *p* = 0.002, sensitivity = 0.58, specificity = 0.90, YI = 48), H-FABP + IGF-BP + cTnI (AUROC = 0.724, 95%CI = 0.593–0.855, *p* = 0.002, sensitivity = 0.54, specificity = 0.93, YI = 0.46), IGF-BP2 + suPAR + cTnI (AUROC = 0.726, 95%CI = 0.600–0.852, *p* = 0.002, sensitivity = 0.54, specificity = 0.93, YI = 0.46), and suPAR + BNP + cTnI (AUROC = 0.689, 95%CI = 0.552–0.826, *p* = 0.013, sensitivity = 0.85, specificity = 0.62, YI = 0.46).

Almost all other combinations also reached a significant *p*-value ≤ 0.050.

### 3.9. Kaplan–Meier Analysis

**Figure 7:**
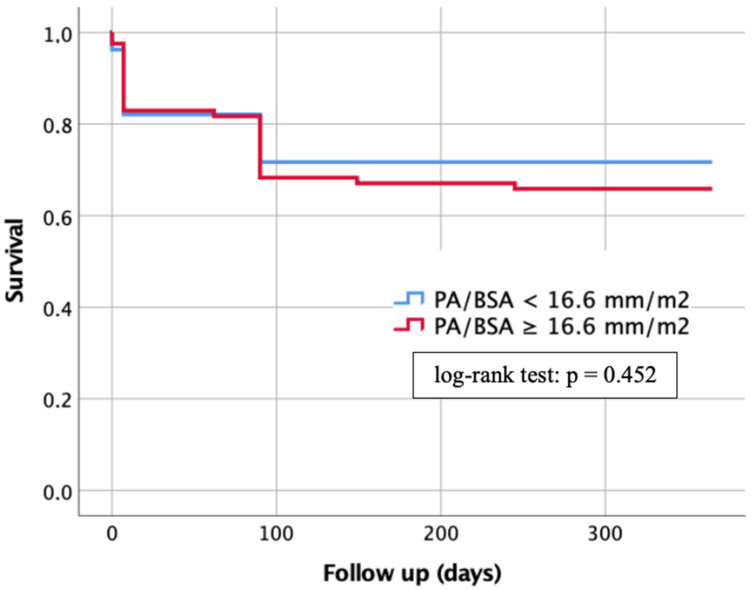
Kaplan–Meier curve for detection of 1-year survival in dependence of a PA/BSA cut-off value ≥ 16.6 mm/m^2^. PA: pulmonary artery; BSA: body surface area.

One-year survival of patients with a PA/BSA < 16.6 mm/m^2^ and patients with a PA/BSA ≥ 16.6 mm/m^2^ was compared using Kaplan–Meier curves, as shown in Figure 7.

Patients with a PA/BSA < 16.6 mm/m^2^ showed a lower risk of death during the 1-year follow-up period at 28.3%, whereas patients with a PA/BSA ≥ 16.6 mm/m^2^ demonstrated a death rate of 34.1%. However, the log-rank test did not show any significant difference with *p* = 0.452.

## 4. Discussion

This study aimed to investigate the relationship between PA/BSA and different biomarkers in patients with severe AS and co-existing PH, as there are currently few studies exploring this relationship.

### 4.1. Can a PA/BSA ≥ 16.6 mm/m^2^ Be Used as Evidence for PH?

The PA/BSA cut-off value of 16.6 mm/m^2^ used in this study was determined using different sPAP values (40–45–50 mmHg). As the current literature does not agree on a single diagnostic sPAP cut-off value for PH diagnosis, to calculate a PA/BSA cut-off, AUROC analyses of the different sPAP values were performed and the rounded average of the results was used [3,4,5,6,7,8]. This value is also closely reflected in a study conducted by Sudo et al. looking at a study population of 770 patients and concluded that a PA/BSA ≥ 1.68 cm/m^2^ (16.8 mm/m^2^) is associated with pre- and post-TAVR PH as well as right heart failure [22]. Other reports also shown that PA dilation is prominent in PH [11,23]. In general, these previous studies show that PA/BSA could provide additional information regarding the risk of death in patients undergoing TAVR. It should be mentioned, however, that severe AS alone may lead to widening of the PA diameter due to vascular remodeling caused by hemodynamic changes. Although PA/BSA tries to consider the variability of the PA diameter in different body types by taking BSA into account, this value alone might not be enough to accurately diagnose PH [23]. Therefore, this study mainly aimed to evaluate the value of different biomarkers for this important task of diagnosing PH pre-TAVR.

### 4.2. Can a PA/BSA ≥ 16.6 mm/m^2^ Be Used as a Prognostic Factor?

Luçon et al. postulated a pre-interventional sPAP ≥ 40 mmHg to be an independent prognostic factor for patients with severe AS undergoing TAVR, regarding their 1-year risk of death [24]. In this work, for an sPAP ≥ 40 mmHg, a PA/BSA cut-off value of 16.6 mm/m^2^ was calculated. Compared to the results found in the study investigating the prognostic value of sPAP ≥ 40 mmHg, the Kaplan–Meier calculation conducted in this study did not yield a significant difference in 1-year mortality between patients with a PA/BSA < 16.6 mm/m^2^ and patients with a PA/BSA ≥ 16.6 mm/m^2^ after TAVR. However, a comparable study conducted by Sudo et al. found a significant difference in mortality between patient groups classified by large or small PA/BSA. Patients with PA/BSA ≥ 16.8 mm/m^2^ showed a cumulative 2-year mortality of 30.0%, compared to a 2-year mortality of 13.7% in the other group [22]. Compared to our work, this study evaluated the two-year mortality as opposed to the one-year mortality, with a slightly different PA/BSA cut-off value of 16.8 mm/m^2^ as opposed to 16.6 mm/m^2^. Moreover, the patient cohort used was considerably larger than ours, with 770 patients compared to 188 patients. These results lead us to believe that PA/BSA might be useful as a prognostic factor before TAVR; however, to confirm this assumption, more research would be necessary.

### 4.3. Can a PA/BSA ≥ 16.6 mm/m^2^ Be Used Together with Biomarkers to Gain Additional Information?

Due to the unconvincing results of the Kaplan–Meier calculation, we combined the data collected through CT-angiography with certain biomarkers obtained through pre-interventional laboratory measurements.

Matching the results of previous studies investigating the levels of BNP or NT-proBNP in patients with PH, our work also found significantly increased levels of BNP/NT-proBNP in patients with PA/BSA ≥ 16.6 mm/m^2^ [25,26,27]. In the available literature, however, PH was defined by sPAP values obtained through echocardiography [27,28] or right heart catheter measurements [26]. Considering the known stimuli for the release of BNP, which encompass cardiovascular diseases such as severe AS, which leads to chronic hypertrophy of the left ventricle, the worth of the calculated cut-off value for BNP (1172.0 pg/mL, sensitivity = 0.51, specificity = 0.80) remains questionable [29].

Another significantly elevated cardiovascular biomarker was H-FABP, an organotropic biomarker which is mainly secreted by cardiomyocytes [30]. Compared to a previous study with patients suffering from severe AS, where concomitant PH was defined by an sPAP ≥ 40 mmHg, no significantly different expression level of H-FABP could be observed in patients “with” and “without” PH [31]. In a recently published study of our working group, this apparent contradiction was explained by the ability of CT angiography to assess vascular geometry and therefore give indirect information about right ventricular strain [32].

Other novel cardiovascular biomarkers such as GDF-15 and IGF-BP2 also showed significantly elevated levels in patients with a PA/BSA ≥ 16.6 mm/m^2^. GDF-15 is a stress-induced cytokine which acts at multiple different sites in the body, as well as a cell-autonomous regulator associated with senescence and apoptosis. It has been previously found to be a predictive biomarker of all-cause mortality and adverse cardiovascular events in pulmonary critical care [33]. GDF-15 levels have also been previously associated with increased mean pulmonary artery pressure, which would, like the results of our study, mean levels would be elevated in PH [34]. Similarly, IGF-BP2, a biomarker which potentially plays a role in the pathobiology of PH, has been previously associated with worse outcomes in pediatric PH patients. It has even been proposed as a potential mechanistic target in pediatric PH [35].

## 5. Conclusions

Although PA/BSA aims to reduce the bias of the PA value caused by different body compositions and sizes, it is still a controversial parameter for diagnosing PH. Combining the parameter with different cardiovascular biomarkers did not lead to a significant increase in the diagnostic precision for detecting PH in patients with severe AS.

## 6. Limitation

The present study is based on data from a small cohort of two medical centers in Austria over a circumscribed time period (2016–2018). Biomarker levels were only measured at baseline without statements regarding expression after TAVR procedure. Additionally, technical pitfalls in echocardiographic and radiological measurements which lead to misclassifications should always be conceded, even if examinations (echocardiography and computed tomography measurements) were performed by experienced clinical investigators.

## Figures and Tables

**Table 1:** Overview of the calculation of the estimated RAP.

IVC Diameter	Respiratory Caliber Fluctuation	RAP
≥21 mm	<50%	15 mmHg
Not corresponding to these constellations		8 mmHg
<21 mm	≥50%	3 mmHg

**Table 2:** Characteristics of the study cohort.

	Overall Cohort n = 188		PA/BSA < 16.6 mm/m^2^ n = 107		PA/BSA ≥ 16.6 mm/m^2^ n = 81		
Clinical Data							*p*-value
Age (years)—mean ± SD	82.8	4.9	82.0	5.0	83.0	4.5	0.139
Gender (male)—%	52.7		60.7		42.0		0.011
Weight (kg)—mean ± SD	74.2	14.6	76.0	15.5	71.0	12.8	0.021
Height (cm)—mean ± SD	166.9	9.1	168.6	8.7	164.7	9.0	0.003
BMI (kg/m^2^)—mean ± SD	26.6	5.3	26.8	5.7	26.3	4.6	0.517
NYHA—median ± IQR	3.0	1.0	3.0	1.0	3.0	1.0	0.277
STSScore—mean ± SD	2.9	1.6	2.4	1.0	3.5	1.8	0.005
Concomitant Disease							*p*-value
Diabetes mellitus—%	21.8		23.4		19.8		0.553
Hypertension—%	79.3		82.2		75.3		0.246
CVD-%	73.4		80.4		64.2		0.025
CVD-1 vessel—%	22.3		25.2		18.5		0.399
CVD-2 vessels—%	9.0		8.4		9.9		0.610
CVD-3 vessels—%	9.6		11.2		7.4		0.469
Myocardial infarction—%	3.2		2.8		3.7		0.737
Atrial fibrillation—%	33.5		29.0		39.5		0.130
Pacemaker—%	7.4		6.5		8.6		0.587
Malignancy—%	21.3		24.3		17.3		0.244
Stroke—%	4.8		3.7		6.2		0.436
PAD—%	5.3		4.7		6.2		0.650
COPD—%	9.0		8.4		9.9		0.729
Echocardiography							*p*-value
LVEF (%)—mean ± SD	55.8	11.0	57.5	9.4	53.4	12.4	0.015
LVEDD (mm)—mean ± SD	46.0	6.1	4.5	0.5	4.6	0.7	0.277
IVSd (mm)—mean ± SD	15.2	2.9	15.0	2.8	15.5	3.2	0.377
AV Vmax (m/s)—mean ± SD	4.3	0.6	4.3	0.5	4.3	0.6	0.678
AVdPmean (mmHg)—mean ± SD	48.6	12.5	48.6	12.0	48.7	13.3	0.949
AVdPmax (mmHg)—mean ± SD	77.8	19.2	77.9	18.3	77.7	20.4	0.950
TAPSE (mm)—mean ± SD	22.1	3.9	22.7	4.1	21.1	3.6	0.077
sPAP (mmHg)—mean ± SD	43.3	15.4	38.5	14.3	49.0	14.8	<0.001
AVI ≥ II°—%	16.0		11.2		22.2		0.053
MVI ≥ II°—%	19.7		15.9		24.7		0.147
TVI ≥ II°—%	12.2		9.3		16.0		0.154
Laboratory data							*p*-value
Creatinine (mg/dL)—median ± IQR	1.0	0.4	1.0	0.3	1.0	0.5	0.694
BNP (pg/mL)—median ± IQR	1174.0	2094.4	823.1	1557.3	2270.5	3263.7	<0.001
cTnI (pg/mL)—median ± IQR	23.5	19.0	21.0	14.0	27.0	22.0	0.055
hsTN (pg/mL)—median ± IQR	2.5	2.3	2.4	2.3	2.7	1.3	0.747
Hkt (%)—median ± IQR	38.4	6.2	39.1	5.3	37.4	7.7	0.013
Hb (g/dL)—median ± IQR	12.8	2.3	13.1	2.4	12.6	2.9	0.011
CK (U/L)—median ± IQR	79.0	68.0	86.5	78.0	75.0	66.0	0.218
sST2 (pg/mL)—median ± IQR	13,947.3	9073.6	14,399.6	10,086.8	13,806.6	6727.2	0.323
GDF-15 (pg/mL)—median ± IQR	922.8	707.5	840.1	577.7	1055.2	778.9	0.040
H-FABP (ng/mL)—median ± IQR	1.3	1.8	1.1	1.5	1.6	1.9	0.007
IGF-BP2 (pg/mL)—median ± IQR	178,153.7	134,892.6	156,947.6	115,637.5	225,232.1	180,916.1	0.005
suPAR (pg/mL)—median ± IQR	3524.1	1740.2	3449.2	1775.6	3767.5	1895.8	0.097

PA: pulmonary artery; BSA: body surface area; BMI: body mass index; CVD: cardiovascular disease; LVEF: left ventricular ejection fraction; LVEDD: left ventricular end-diastolic diameter; IVSd: interventricular septal thickness at diastole; AV Vmax: maximal velocity over aortic valve; AV dpmean: mean pressure gradient over aortic valve; AV dpmax: maximal pressure gradient over aortic valve; TAPSE: tricuspid annular plane systolic excursion; sPAP: systolic pulmonary artery pressure; AVI: aortic valve insufficiency; MVI: mitral valve insufficiency; TVI: tricuspid valve insufficiency; BNP: brain natriuretic peptide; cTnI: cardiac troponin I; hsTN: high-sensitive troponin; Hkt: hematocrit; Hb: hemoglobin; CK: creatine kinase; sST2: soluble suppression of tumorigenesis-2; GDF-15: growth/differentiation factor-15; H-FABP: heart-type fatty acid-binding protein; IGF-BP2: insulin-like growth factor binding protein 2; suPAR: soluble urokinase-type plasminogen activator receptor; SD: standard deviation; IQR: interquartile range.

**Table 3:** Tabular overview of AUROC analyses of PA/AA-Ratio for prediction of sPAP ≥ 40, 45, and 50 mmHg with concerning cut-off values, Youden Index, sensitivity, and specificity.

Value	Prediction	AUC	95%CI	*p*-Value	Cut-Off	Sensitivity	Specificity	Youden Index
PA/BSA (mm/m^2^)	sPAP ≥ 40 mmHg	0.741	0.656–0.826	<0.001	16.53	0.62	0.79	0.41
PA/BSA (mm/m^2^)	sPAP ≥ 45 mmHg	0.642	0.584–0.761	<0.001	16.65	0.62	0.71	0.33
PA/BSA (mm/m^2^)	sPAP ≥ 50 mmHg	0.742	0.658–0.826	<0.001	16.65	0.74	0.68	0.42

sPAP: systolic pulmonary artery pressure; PA: pulmonary artery; BSA: body surface area.

**Table 4:** Tabular overview of AUROC analyses of singular biomarkers for prediction of a PA/BSA value ≥ 16.6 mm/m^2^ with concerning Youden Index, sensitivity, and specificity.

Value	Prediction	AUC	95%CI	*p*-Value	Cut-Off	Sensitivity	Specificity	Youden Index
sST2 (pg/mL)	PA/BSA ≥ 16.6 mm/m^2^	0.466	0.382–0.550	0.433	11,545.5	0.72	0.33	0.05
GDF-15 (pg/mL)	PA/BSA ≥ 16.6 mm/m^2^	0.595	0.509–0.680	0.029	1172.0	0.44	0.76	0.20
H-FABP (ng/mL)	PA/BSA ≥ 16.6 mm/m^2^	0.616	0.533–0.700	0.080	1.6	0.54	0.69	0.23
IGF-BP2 (pg/mL)	PA/BSA ≥ 16.6 mm/m^2^	0.694	0.570–0.818	0.050	198,440.4	0.63	0.70	0.33
suPAR (pg/mL)	PA/BSA ≥ 16.6 mm/m^2^	0.572	0.487–0.656	0.097	2753.9	0.83	0.31	0.14
BNP (pg/mL)	PA/BSA ≥ 16.6 mm/m^2^	0.684	0.604–0.764	<0.001	2194.0	0.51	0.80	0.32
hsTN (pg/mL)	PA/BSA ≥ 16.6 mm/m^2^	0.518	0.410–0.626	0.748	2.0	0.76	0.39	0.15
cTNI (pg/mL)	PA/BSA ≥ 16.6 mm/m^2^	0.635	0.501–0.770	0.055	24.5	0.66	0.66	0.31

PA: pulmonary artery; BSA: body surface area; BNP: brain natriuretic peptide; cTnI: cardiac troponin I; hsTN: high-sensitive troponin; sST2: soluble suppression of tumorigenesis-2; GDF-15: growth/differentiation factor-15; H-FABP: heart-type fatty acid-binding protein; IGF-BP2: insulin-like growth factor binding protein 2; suPAR: soluble urokinase-type plasminogen activator receptor.

**Table 5:** Tabular overview of AUROC analyses of a combination of 2 biomarkers for prediction of a PA/BSA value ≥ 16.6 mm/m^2^ with concerning Youden Index, sensitivity, and specificity.

Values	Prediction	AUC	95%CI	*p*-Value	Sensitivity	Specificity	Youden Index
sST2—GDF-15	PA/BSA ≥ 16.6 mm/m^2^	0.600	0.514–0.686	0.022	0.34	0.88	0.22
sST2—H-FABP	PA/BSA ≥ 16.6 mm/m^2^	0.625	0.543–0.708	0.004	0.67	0.56	0.23
sST2—IGF-BP2	PA/BSA ≥ 16.6 mm/m^2^	0.700	0.577–0.823	0.004	0.53	0.81	0.35
sST2—suPAR	PA/BSA ≥ 16.6 mm/m^2^	0.599	0.515–0.682	0.220	0.30	0.93	0.22
sST2—BNP	PA/BSA ≥ 16.6 mm/m^2^	0.706	0.626–0.785	< 0.001	0.71	0.67	0.38
sST2—hsTN	PA/BSA ≥ 16.6 mm/m^2^	0.524	0.415–0.634	0.056	0.77	0.38	0.14
sST2—cTnI	PA/BSA ≥ 16.6 mm/m^2^	0.619	0.484–0.754	0.092	0.59	0.66	0.25
GDF-15—H-FABP	PA/BSA ≥ 16.6 mm/m^2^	0.619	0.534–0.704	0.006	0.67	0.62	0.29
GDF-15—IGF-BP2	PA/BSA ≥ 16.6 mm/m^2^	0.698	0.570–0.825	0.005	0.64	0.72	0.36
GDF-15—suPAR	PA/BSA ≥ 16.6 mm/m^2^	0.609	0.525–0.692	0.013	0.49	0.74	0.22
GDF-15—BNP	PA/BSA ≥ 16.6 mm/m^2^	0.682	0.599–0.765	<0.001	0.76	0.58	0.34
GDF-15—hsTN	PA/BSA ≥ 16.6 mm/m^2^	0.580	0.468–0.693	0.153	0.51	0.74	0.25
GDF-15—cTnI	PA/BSA ≥ 16.6 mm/m^2^	0.636	0.498–0.774	0.059	0.48	0.83	0.31
H-FABP—IGF-BP2	PA/BSA ≥ 16.6 mm/m^2^	0.694	0.567–0.821	0.006	0.46	0.88	0.35
H-FABP—suPAR	PA/BSA ≥ 16.6 mm/m^2^	0.612	0.527–0.697	0.010	0.51	0.74	0.25
H-FABP—BNP	PA/BSA ≥ 16.6 mm/m^2^	0.698	0.617–0.778	<0.001	0.78	0.62	0.39
H-FABP—hsTN	PA/BSA ≥ 16.6 mm/m^2^	0.606	0.497–0.715	0.056	0.45	0.77	0.22
H-FABP—cTnI	PA/BSA ≥ 16.6 mm/m^2^	0.624	0.486–0.762	0.086	0.59	0.68	0.28
IGF-BP2—suPAR	PA/BSA ≥ 16.6 mm/m^2^	0.684	0.559–0.810	0.008	0.43	0.88	0.32
IGF-BP2—BNP	PA/BSA ≥ 16.6 mm/m^2^	0.721	0.585–0.857	0.004	0.68	0.73	0.41
IGF-BP2—cTnI	PA/BSA ≥ 16.6 mm/m^2^	0.694	0.570–0.818	0.005	0.63	0.70	0.33
suPAR—BNP	PA/BSA ≥ 16.6 mm/m^2^	0.695	0.615–0.775	<0.001	0.47	0.84	0.31
suPAR—hsTN	PA/BSA ≥ 16.6 mm/m^2^	0.572	0.487–0.656	0.097	0.83	0.31	0.14
suPAR—cTnI	PA/BSA ≥ 16.6 mm/m^2^	0.669	0.539–0.798	0.017	0.69	0.63	0.32
BNP—hsTN	PA/BSA ≥ 16.6 mm/m^2^	0.700	0.603–0.798	<0.001	0.54	0.82	0.36
BNP—cTnI	PA/BSA ≥ 16.6 mm/m^2^	0.680	0.544–0.816	0.018	0.89	0.44	0.33

PA: pulmonary artery; BSA: body surface area; BNP: brain natriuretic peptide; cTnI: cardiac troponin I; hsTN: high-sensitive troponin; sST2: soluble suppression of tumorigenesis-2; GDF-15: growth/differentiation factor-15; H-FABP: heart-type fatty acid-binding protein; IGF-BP2: insulin-like growth factor binding protein 2; suPAR: soluble urokinase-type plasminogen activator receptor.

**Table 6:** Tabular overview of AUROC analyses of a combination of 3 biomarkers for prediction of a PA/BSA value ≥ 16.6 mm/m^2^ with concerning Youden Index, sensitivity, and specificity.

Values	Prediction	AUC	95%CI	*p*-Value	Sensitivity	Specificity	Youden Index
sST2—GDF-15—H-FABP	PA/BSA ≥ 16.6 mm/m^2^	0.633	0.550–0.717	0.002	0.55	0.71	0.26
sST2—GDF-15—IGF-BP2	PA/BSA ≥ 16.6 mm/m^2^	0.702	0.575–0.829	0.004	0.82	0.53	0.36
sST2—GDF-15—suPAR	PA/BSA ≥ 16.6 mm/m^2^	0.614	0.529–0.699	0.009	0.40	0.87	0.26
sST2—GDF-15—BNP	PA/BSA ≥ 16.6 mm/m^2^	0.708	0.627–0.788	<0.001	0.75	0.62	0.36
sST2—GDF-15—hsTN	PA/BSA ≥ 16.6 mm/m^2^	0.584	0.473–0.695	0.136	0.36	0.85	0.21
sST2—GDF-15—cTnI	PA/BSA ≥ 16.6 mm/m^2^	0.637	0.496–0.778	0.058	0.48	0.85	0.33
sST2—H-FABP—IGF-BP2	PA/BSA ≥ 16.6 mm/m^2^	0.719	0.595–0.844	0.002	0.68	0.72	0.40
sST2—H-FABP—suPAR	PA/BSA ≥ 16.6 mm/m^2^	0.630	0.548–0.712	0.003	0.61	0.61	0.22
sST2—H-FABP—BNP	PA/BSA ≥ 16.6 mm/m^2^	0.714	0.635–0.793	<0.001	0.73	0.65	0.38
sST2—H-FABP—hsTN	PA/BSA ≥ 16.6 mm/m^2^	0.619	0.512–0.725	0.035	0.68	0.56	0.24
sST2—H-FABP—cTnI	PA/BSA ≥ 16.6 mm/m^2^	0.626	0.487–0.765	0.080	0.67	0.61	0.28
sST2—IGF-BP2—suPAR	PA/BSA ≥ 16.6 mm/m^2^	0.697	0.573–0.821	0.004	0.53	0.81	0.35
sST2—IGF-BP2—BNP	PA/BSA ≥ 16.6 mm/m^2^	0.727	0.593–0.862	0.003	0.64	0.79	0.43
sST2—IGF-BP2—cTnI	PA/BSA ≥ 16.6 mm/m^2^	0.731	0.605–0.858	0.001	0.64	0.80	0.44
sST2—suPAR—BNP	PA/BSA ≥ 16.6 mm/m^2^	0.698	0.618–0.778	<0.001	0.60	0.74	0.34
sST2—suPAR—hsTN	PA/BSA ≥ 16.6 mm/m^2^	0.559	0.449–0.669	0.294	0.57	0.61	0.18
sST2—suPAR—cTnI	PA/BSA ≥ 16.6 mm/m^2^	0.699	0.574–0.823	0.005	0.76	0.66	0.42
sST2—BNP—hsTN	PA/BSA ≥ 16.6 mm/m^2^	0.708	0.609–0.807	<0.001	0.62	0.74	0.36
sST2—BNP—cTnI	PA/BSA ≥ 16.6 mm/m^2^	0.688	0.553–0.823	0.013	0.81	0.53	0.34
GDF-15—H-FABP—IGF-BP2	PA/BSA ≥ 16.6 mm/m^2^	0.698	0.570–0.825	0.005	0.57	0.79	0.36
GDF-15—H-FABP—suPAR	PA/BSA ≥ 16.6 mm/m^2^	0.627	0.544–0.711	0.003	0.75	0.51	0.26
GDF-15—H-FABP—BNP	PA/BSA ≥ 16.6 mm/m^2^	0.699	0.619–0.779	<0.001	0.82	0.54	0.36
GDF-15—H-FABP—hsTN	PA/BSA ≥ 16.6 mm/m^2^	0.610	0.499–0.720	0.051	0.70	0.64	0.34
GDF-15—H-FABP—cTnI	PA/BSA ≥ 16.6 mm/m^2^	0.635	0.496–0.774	0.061	0.48	0.83	0.31
GDF-15—IGF-BP2—suPAR	PA/BSA ≥ 16.6 mm/m^2^	0.695	0.567–0.823	0.006	0.50	0.86	0.36
GDF-15—IGF-BP2—BNP	PA/BSA ≥ 16.6 mm/m^2^	0.727	0.590–0.864	0.004	0.74	0.67	0.41
GDF-15—IGF-BP2—cTnI	PA/BSA ≥ 16.6 mm/m^2^	0.732	0.600–0.863	0.002	0.58	0.90	0.48
GDF-15—suPAR—BNP	PA/BSA ≥ 16.6 mm/m^2^	0.699	0.619–0.779	<0.001	0.69	0.64	0.32
GDF-15—suPAR—hsTN	PA/BSA ≥ 16.6 mm/m^2^	0.594	0.486–0.702	0.095	0.72	0.49	0.21
GDF-15—suPAR—cTnI	PA/BSA ≥ 16.6 mm/m^2^	0.653	0.516–0.790	0.034	0.63	0.76	0.39
GDF-15—BNP—hsTN	PA/BSA ≥ 16.6 mm/m^2^	0.707	0.607–0.806	<0.001	0.51	0.85	0.36
GDF-15—BNP—cTnI	PA/BSA ≥ 16.6 mm/m^2^	0.648	0.500–0.797	0.056	0.71	0.65	0.36
H-FABP—IGF-BP2—suPAR	PA/BSA ≥ 16.6 mm/m^2^	0.694	0.567–0.820	0.006	0.43	0.91	0.34
H-FABP—IGF-BP2—BNP	PA/BSA ≥ 16.6 mm/m^2^	0.721	0.580–0.861	0.005	0.52	0.91	0.43
H-FABP—IGF-BP2—cTnI	PA/BSA ≥ 16.6 mm/m^2^	0.724	0.593–0.855	0.002	0.54	0.93	0.46
H-FABP—suPAR—BNP	PA/BSA ≥ 16.6 mm/m^2^	0.698	0.618–0.778	<0.001	0.82	0.55	0.37
H-FABP—suPAR—hsTN	PA/BSA ≥ 16.6 mm/m^2^	0.627	0.518–0.737	0.024	0.68	0.62	0.30
H-FABP—suPAR—cTnI	PA/BSA ≥ 16.6 mm/m^2^	0.668	0.536–0.801	0.019	0.82	0.51	0.33
H-FABP—BNP—hsTN	PA/BSA ≥ 16.6 mm/m^2^	0.732	0.638–0.826	<0.001	0.87	0.56	0.43
H-FABP—BNP—cTnI	PA/BSA ≥ 16.6 mm/m^2^	0.669	0.527–0.811	0.029	0.75	0.65	0.40
IGF-BP2—suPAR—BNP	PA/BSA ≥ 16.6 mm/m^2^	0.720	0.584–0.856	0.004	0.52	0.88	0.40
IGF-BP2—suPAR—cTnI	PA/BSA ≥ 16.6 mm/m^2^	0.726	0.600–0.852	0.002	0.54	0.93	0.46
IGF-BP2—BNP—cTnI	PA/BSA ≥ 16.6 mm/m^2^	0.733	0.599–0.868	0.003	0.56	0.88	0.44
suPAR—BNP—hsTN	PA/BSA ≥ 16.6 mm/m^2^	0.710	0.612–0.809	<0.001	0.70	0.66	0.36
suPAR—BNP—cTnI	PA/BSA ≥ 16.6 mm/m^2^	0.689	0.552–0.826	0.013	0.85	0.62	0.46

PA: pulmonary artery; BSA: body surface area; BNP: brain natriuretic peptide; cTnI: cardiac troponin I; hsTN: high-sensitive troponin; sST2: soluble suppression of tumorigenesis-2; GDF-15: growth/differentiation factor-15; H-FABP: heart-type fatty acid-binding protein; IGF-BP2: insulin-like growth factor binding protein 2; suPAR: soluble urokinase-type plasminogen activator receptor.

## Data Availability

The data presented in this study are available on request from the corresponding author.

## Supplementary Materials

The following supporting information can be downloaded at: https://www.mdpi.com/article/10.3390/jcdd10010022/s1; Figure S1: Overview of calculations of the estimated sample size.

## Abbreviations

AS	aortic stenosis
AUC	area under the curve
AUROC	area under the receiver operator characteristics
BNP	brain natriuretic peptide
BSA	body surface area
cTnI	cardiac troponin I
ESC	European Society of Cardiology
GDF-15	growth/differentiation factor-15
H-FABP	heart-type fatty acid-binding protein
hsTN	high-sensitive troponin
IGF-BP2	insulin-like growth factor binding protein 2
IQR	interquartile range
IVC	inferior vena cava
LVEF	left ventricular ejection fraction
PA	pulmonary artery
PA/BSA	pulmonary artery diameter/body surface area
PH	pulmonary hypertension
RAP	right atrial pressure
SD	standard deviation
sPAP	systolic pulmonary artery pressure
sST2	soluble suppression of tumorigenesis-2
suPAR	soluble urokinase-type plasminogen activator receptor
TRV	tricuspid regurgitant jet velocity
YI	Youden Index

## References

[1] Masri A., Gleason T.G., Lee J.S., Cavalcante J.L. (2019). Pulmonary Hypertension Persistency in Severe Aortic Stenosis Patients Treated With TAVR. JACC Cardiovasc. Imaging.

[2] Alushi B., Beckhoff F., Leistner D., Franz M., Reinthaler M., Stähli B.E., Morguet A., Figulla H.R., Doenst T., Maisano F. (2018). Pulmonary Hypertension in Patients With Severe Aortic Stenosis: Prognostic Impact After Transcatheter Aortic Valve Replacement: Pulmonary Hypertension in Patients Undergoing TAVR. JACC Cardiovasc. Imaging.

[3] Schewel J., Schlüter M., Schmidt T., Kuck K., Frerker C., Schewel D. (2020). Correlation between Doppler echocardiography and right heart catheterization assessment of systolic pulmonary artery pressure in patients with severe aortic stenosis. Echocardiography.

[4] Saraiva R.M., Matsumura Y., Yamano T., Greenberg N., Thomas J.D., Shiota T. (2010). Relation of Left Atrial Dysfunction to Pulmonary Artery Hypertension in Patients With Aortic Stenosis and Left Ventricular Systolic Dysfunction. Am. J. Cardiol..

[5] Nijenhuis V., Huitema M., Vorselaars V., Swaans M., de Kroon T., van der Heyden J., Rensing B., Heijmen R., Berg J.T., Post M. (2016). Echocardiographic pulmonary hypertension probability is associated with clinical outcomes after transcatheter aortic valve implantation. Int. J. Cardiol..

[6] Bishu K., Suri R.M., Nkomo V.T., Kane G.C., Greason K.L., Reeder G.S., Mathew V., Holmes D.R., Rihal C.S., Melduni R.M. (2014). Prognostic Impact of Pulmonary Artery Systolic Pressure in Patients Undergoing Transcatheter Aortic Valve Replacement for Aortic Stenosis. Am. J. Cardiol..

[7] D’Ascenzo F., Conrotto F., Salizzoni S., Rossi M.L., Nijhoff F., Gasparetto V., Barbanti M., Mennuni M., Omedè P., Marra W.G. (2015). Incidence, predictors, and impact on prognosis of systolic pulmonary artery pressure and its improvement after transcatheter aortic valve implantation: A multicenter registry. J. Am. Coll. Cardiol..

[8] Lancellotti P., Magne J., Donal E., O’Connor K., Dulgheru R., Rosca M., Pierard L.A. (2012). Determinants and Prognostic Significance of Exercise Pulmonary Hypertension in Asymptomatic Severe Aortic Stenosis. Circulation.

[9] Durmaz T., Ayhan H., Keleş T., Aslan A.N., Kasapkara H.A., Sari C., Bilen E., Bayram N.A., Akçay M., Bozkurt E. (2014). The Effect of Transcatheter Aortic Valve Implantation on Pulmonary Hypertension. Echocardiography.

[10] Humbert M., Kovacs G., Hoeper M.M., Badagliacca R., Berger R.M.F., Brida M., Carlsen J., Coats A.J.S., Escribano-Subias P., Ferrari P. (2022). 2022 ESC/ERS Guidelines for the diagnosis and treatment of pulmonary hypertension. Eur. Hear. J..

[11] O’Sullivan C.J., Montalbetti M., Zbinden R., Kurz D.J., Bernheim A.M., Liew A., Meyer M.R., Tuller D., Eberli F.R. (2018). Screening For Pulmonary Hypertension With Multidetector Computed Tomography Among Patients With Severe Aortic Stenosis Undergoing Transcatheter Aortic Valve Implantation. Front. Cardiovasc. Med..

[12] Turner V.L., Jubran A., Kim J.B., Maret E., Moneghetti K.J., Haddad F., Amsallem M., Codari M., Hinostroza V., Mastrodicasa D. (2021). CTA pulmonary artery enlargement in patients with severe aortic stenosis: Prognostic impact after TAVR. J. Cardiovasc. Comput. Tomogr..

[13] Wu Y., Pan N., An Y., Xu M., Tan L., Zhang L. (2021). Diagnostic and Prognostic Biomarkers for Myocardial Infarction. Front. Cardiovasc. Med..

[14] Wang X.-Y., Zhang F., Zhang C., Zheng L.-R., Yang J. (2020). The Biomarkers for Acute Myocardial Infarction and Heart Failure. BioMed Res. Int..

[15] Castiglione V., Aimo A., Vergaro G., Saccaro L., Passino C., Emdin M. (2021). Biomarkers for the diagnosis and management of heart failure. Hear. Fail. Rev..

[16] Ibrahim N.E., Januzzi J.L. (2018). Established and Emerging Roles of Biomarkers in Heart Failure. Circ. Res..

[17] Boxhammer E., Mirna M., Bäz L., Bacher N., Topf A., Sipos B., Franz M., Kretzschmar D., Hoppe U.C., Lauten A. (2022). Soluble ST2 as a Potential Biomarker for Risk Assessment of Pulmonary Hypertension in Patients Undergoing TAVR?. Life.

[18] Muessig J.M., Lichtenauer M., Wernly B., Kelm M., Franz M., Bäz L., Schulze P.C., Racher V., Zimmermann G., Figulla H.-R. (2018). Insulin like growth factor binding protein 2 (IGFBP-2) for risk prediction in patients with severe aortic stenosis undergoing Transcatheter Aortic Valve Implantation (TAVI). Int. J. Cardiol..

[19] Hodges G.W., Bang C., Eugen-Olsen J., Olsen M.H., Boman K., Ray S., Kesäniemi A.Y., Jeppesen J.L., Wachtell K. (2018). SuPAR predicts postoperative complications and mortality in patients with asymptomatic aortic stenosis. Open Hear..

[20] Kim J.B., Kobayashi Y., Moneghetti K.J., Brenner D.A., O’Malley R., Schnittger I., Wu J.C., Murtagh G., Beshiri A., Fischbein M. (2017). GDF-15 (Growth Differentiation Factor 15) Is Associated With Lack of Ventricular Recovery and Mortality After Transcatheter Aortic Valve Replacement. Circ. Cardiovasc. Interv..

[21] Mirna M., Wernly B., Paar V., Jung C., Jirak P., Figulla H.R., Kretzschmar D., Franz M., Hoppe U.C., Lichtenauer M. (2018). Multi-biomarker analysis in patients after transcatheter aortic valve implantation (TAVI). Biomarkers: Biochemical indicators of exposure, response, and susceptibility to chemicals. Biomarkers.

[22] Sudo M., Sugiura A., Treiling L., Al-Kassou B., Shamekhi J., Kütting D., Wilde N., Weber M., Zimmer S., Nickenig G. (2021). Baseline PA/BSA ratio in patients undergoing transcatheter aortic valve replacement–A novel CT-based marker for the prediction of pulmonary hypertension and outcome. Int. J. Cardiol..

[23] Eberhard M., Mastalerz M., Pavicevic J., Frauenfelder T., Nietlispach F., Maisano F., Tanner F.C., Nguyen-Kim T. (2017). Value of CT signs and measurements as a predictor of pulmonary hypertension and mortality in symptomatic se-vere aortic valve stenosis. The international journal of cardiovascular imaging. Int. J. Cardiovasc. Imag..

[24] Luçon A., Oger E., Bedossa M., Boulmier D., Verhoye J.P., Eltchaninoff H., Iung B., Leguerrier A., Laskar M., Leprince P. (2014). Prognostic Implications of Pulmonary Hypertension in Patients With Severe Aortic Stenosis Undergoing Transcatheter Aortic Valve Implantation: Study from the FRANCE 2 Registry. Circ. Cardiovasc. Interv..

[25] Gumauskiene B., Padervinskiene L., Vaskelyte J.J., Vaitiekiene A., Lapinskas T., Hoppenot D., Miliauskas S., Galnaitiene G., Simkus P., Ereminiene E. (2019). Left Ventricular Morphology and Function as a Determinant of Pulmonary Hypertension in Patients with Severe Aortic Stenosis: Cardiovascular Magnetic Resonance Imaging Study. Medicina.

[26] Maeder M.T., Weber L., Ammann P., Buser M., Ehl N.F., Gerhard M., Brenner R., Haager P.K., Maisano F., Rickli H. (2020). Relationship between B-type natriuretic peptide and invasive haemodynamics in patients with severe aortic valve stenosis. ESC Hear. Fail..

[27] Calin A., Mateescu A.D., Rosca M., Beladan C.C., Enache R., Botezatu S., Cosei I., Calin C., Simion M., Ginghina C. (2017). Left atrial dysfunction as a determinant of pulmonary hypertension in patients with severe aortic stenosis and preserved left ventricular ejection fraction. Int. J. Cardiovasc. Imaging.

[28] Gumauskienė B., Krivickienė A., Jonkaitienė R., Vaškelytė J.J., Siudikas A., Ereminienė E. (2018). Impact of Left Ventricular Diastolic Dysfunction and Biomarkers on Pulmonary Hypertension in Patients with Severe Aortic Stenosis. Medicina.

[29] Cao Z., Jia Y., Zhu B. (2019). BNP and NT-proBNP as Diagnostic Biomarkers for Cardiac Dysfunction in Both Clinical and Forensic Medicine. Int. J. Mol. Sci..

[30] Ye X.-D., He Y., Wang S., Wong G.T., Irwin M., Xia Z. (2018). Heart-type fatty acid binding protein (H-FABP) as a biomarker for acute myocardial injury and long-term post-ischemic prognosis. Acta Pharmacol. Sin..

[31] Boxhammer E., Köller C., Paar V., Fejzic D., Rezar R., Reiter C., Kammler J., Kellermair J., Hammerer M., Blessberger H. (2022). Systolic Pulmonary Artery Pressure and Cardiovascular Biomarkers—New Non-Invasive Ways to Detect Pulmonary Hypertension in Patients with Severe Aortic Valve Stenosis Undergoing TAVR?. Rev. Cardiovasc. Med..

[32] Boxhammer E., Paar V., Jirak P., Köller C., Demirel O., Eder S., Reiter C., Kammler J., Kellermair J., Hammerer M. (2022). Main pulmonary artery diameter in combination with cardiovascular biomarkers. New possibilities to identify pulmonary hypertension in patients with severe aortic valve stenosis?. Minerva Medica.

[33] Verhamme F.M., Freeman C.M., Brusselle G.G., Bracke K.R., Curtis J.L. (2019). GDF-15 in Pulmonary and Critical Care Medicine. Am. J. Respiratory Cell Mol. Biol..

[34] Larissi K., Politou M., Margeli A., Poziopoulos C., Flevari P., Terpos E., Papassotiriou I., Voskaridou E. (2019). The Growth Differentiation Factor-15 (GDF-15) levels are increased in patients with compound heterozygous sickle cell and beta-thalassemia (HbS/βthal), correlate with markers of hemolysis, iron burden, coagulation, endothelial dysfunction and pulmonary hypertension. Blood Cells Mol. Dis..

[35] Griffiths M., Yang J., Nies M., Vaidya D., Brandal S., Williams M., Matsui E.C., Grant T., Damico R., Ivy D. (2020). Pediatric pulmonary hypertension: Insulin-like growth factor-binding protein 2 is a novel marker associated with disease severity and survival. Pediatr. Res..

