# Supplementary material for: A Story of PA/BSA and Biomarkers to Diagnose Pulmonary Hypertension in Patients with Severe Aortic Valve Stenosis—The Rise of IGF-BP2 and GDF-15
Source: J Cardiovasc Dev Dis. 2023 Jan 5;10(1):22. doi: 10.3390/jcdd10010022 (PMC9864369; doi:10.3390/jcdd10010022)
Supplement: Supplementary file 1 [file jcdd-10-00022-s001.zip › jcdd-2126575-supplementary.pdf]

Estimation of sample size

Formula:

$$\textbf{Estimated sample size} = \frac{z^2 * p(1-p)}{\varepsilon^2}$$

z = confidence level
p = sample proportion
ε = margin of error

Version 1: Estimated sample size = 385
- z = 1.96
- p = 0.5
- ε = 0.05

Version 2: Estimated sample size = 273
- z = 1.65
- p = 0.5
- ε = 0.05

Version 3: Estimated sample size = 97
- z = 1.96
- p = 0.5
- ε = 0.10

Version 4: Estimated sample size = 69
- z = 1.65
- p = 0.5
- ε = 0.10

Average of all versions: Estimated sample size = 206
